# Polymeric Hydrogelator-Based Molecular Gels Containing Polyaniline/Phosphoric Acid Systems

**DOI:** 10.3390/gels8080469

**Published:** 2022-07-27

**Authors:** Yutaka Ohsedo, Mayumi Sasaki

**Affiliations:** 1 Division of Engineering, Faculty of Engineering, Nara Women’s University, Kitauoyahigashi-machi, Nara 630-8506, Japan; 2 Graduate School of Human Centered Engineering, Nara Women’s University, Kitauoyahigashi-machi, Nara 630-8506, Japan; vam_sasaki@cc.nara-wu.ac.jp

**Keywords:** molecular gels, polymer gelators, thixotropic behavior, polyanilines, electrode materials

## Abstract

To expand the range of applications of hydrogels, researchers are interested in developing novel molecular hydrogel materials that have affinities for the living body and the ability to mediate electrical signals. In this study, a simple mixing method for creating a novel composite molecular gel is employed, which combines a hydrophilic conductive polymer, a polyaniline/phosphoric acid complex, and a polymer hydrogelator as a matrix. The composite hydrogel showed an improved gel-forming ability; more effective mechanical properties, with an increased strain value at the sol–gel transition point compared to the single system, which may be sufficient for paintable gel; and a better electrochemical response, due to the electrically conducting polyaniline component. These findings demonstrate the applicability of the new composite hydrogels to new potential paintable electrode materials.

## 1. Introduction

Electrically conducting polymers have been actively researched and developed as active materials for thin-film electronic devices, taking advantage of their light weight and thin-film formation capabilities, which are the characteristics of organic polymers [1,2,3,4,5,6]. Additionally, good solubility or dispersity in solvents is important in preparing thin films of conductive polymers [1,2,3,4,5,6,7,8,9].

Polyaniline has long been known as an insoluble material, aniline black, and its use as an active material for functional electrodes continues to be actively investigated [10,11]. More recently, water-dispersed polyaniline particles have become useful materials with excellent maneuverability that can guarantee electronic and electrical conductivity after film formation [11,12]. On the other hand, water-soluble polyaniline, in which water-soluble functional groups have been introduced into the molecular structure of the polymeric main chain, is another useful material that exhibits electrical conductivity after film formation [1,11,13]. The above water-soluble or hydrophilic conductive polymers have a good affinity for hydrogels [14,15,16,17,18,19,20,21], which comprise new soft materials, and their successful complexation with the hydrogel matrix allows us to obtain a gel state that is difficult to obtain with a conductive polymer alone. Since this soft electron-conductive composite material is expected to have a high affinity for the living body, which is a hydrogel, it is expected to be used as a novel gel material for sensing devices and artificial skin in direct contact with the body.

Recently, molecular gels [22,23,24,25,26,27,28,29,30,31,32,33,34,35,36,37] composed of low-molecular-weight gelators (LMWGs) or polymer gelators [38,39,40] have received attention as advanced soft materials with functionalities such as stimuli responsiveness [37] and thixotropy [22,23,24,41] (mechanically induced reversible sol-to-gel and gel-to-sol transition behavior). Additionally, molecular gels composed of multicomponent LMWGs have been investigated to create molecular gels with new properties [42,43,44,45,46,47,48]. Previously, we employed a simple mixing method to create multicomponent molecular gels with improved properties, which were composed of LMWG homologs [29,30,49,50]. Additionally, by mixing the hydrogelator and synthetic clay, the composite hydrogels showed enhanced thixotropic behavior [51].

**Scheme 1:**
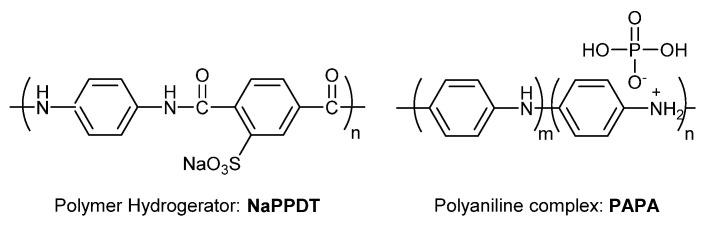
Chemical structures of the polymer hydrogelator (**NaPPDT**) and water-soluble polyaniline complex (**PAPA**).

To develop novel gel materials that are expected to have an affinity with living organisms and that can mediate electrical signals, this study aimed to generate conductive molecular hydrogels by compositing hydrophilic conductive polymers through mixing, using a polymeric hydrogelator as a matrix to form the hydrogel material. Poly(sodium-3–sulfo-p-phenyleneterephthalamide) (**NaPPDT**) [52] was selected as a polymer hydrogelator (Scheme 1), as it is capable of creating various composite materials, such as LAPONITE^®^ [53], a water-soluble silicate, and polyaniline/water-soluble polymer polyion complexes [54], with better gel properties. In this study, a hydrophilic conductive polymer complexed with phosphoric acid (**PAPA**, Scheme 1) was selected as the hydrophilic conductive polymer, as it enabled us to obtain water-soluble conductive polyaniline polymerized from aqueous aniline phosphoric acid solutions through oxidation. Polyaniline with phosphoric acid or phosphoric acid moiety as a dopant has been reported as a better combination for conducting polymers [18,55,56]. Molecular polymer hydrogels composed of **NaPPDT** and aqueous solutions of the polyaniline complex of **PAPA** (mixed-system molecular gels) were prepared, and their mechanical properties were evaluated to determine whether simple mixing can produce conductive molecular gel materials with an electrochemical response while maintaining the properties of the molecular gels. Such electrochemically active composite gels are expected to become a paintable electrode material, facilitating the fabrication of electrochemical devices such as glucose sensors on a variety of media, e.g., cloths or papers [18].

## 2. Results and Discussion

**Figure 1:**
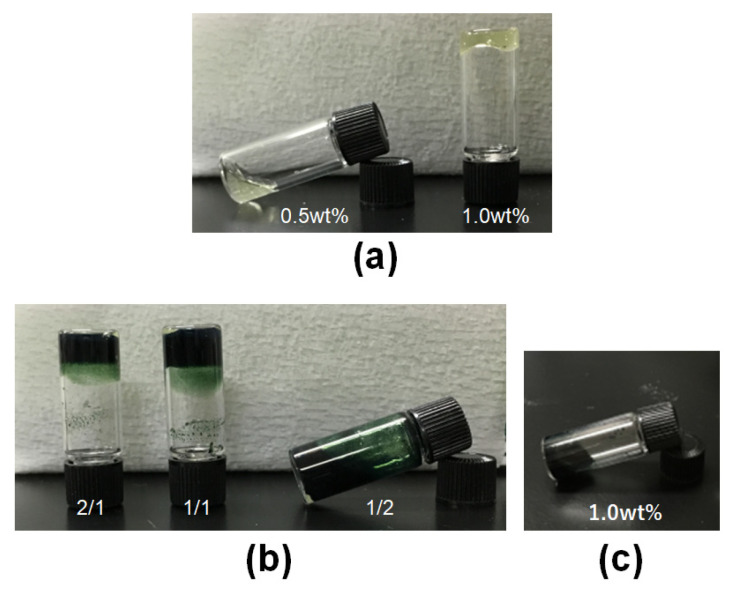
Photos of gelation test: (**a**) **NaPPDT** aqueous solutions in vials, (**b**) 1.0 wt% **NaPPDT**/1.0 wt% **PAPA** aq. mixed in vials, and (**c**) 1.0 wt% **PAPA** aq.

**PAPA** was prepared from an oxidative polymerized aniline in phosphoric acid aqueous solution, and, after purification through dialysis, it yielded a polyaniline complex aqueous solution (**PAPA** aq.) with a dark greenish color. The concentration of **PAPA** aq. was fixed at 1.0 wt% in the dry weight standard of **PAPA**. A mixture of **NaPPDT**/**PAPA** was produced using a vortex mixer (Figure 1), with varied compositions of 1.0 wt% **NaPPDT** hydrogel aqueous solution and 1.0 wt% **PAPA** aqueous solution. The gel formation ability of the composite gels was evaluated using the vial inversion method. After mixing, the concentration of **NaPPDT** became 0.5 wt%, which showed a liquid state in its single solution (Figure 1a), and the composite hydrogel **NaPPDT**/**PAPA** 1/1 (*w*/*w*) exhibited a gel state (Figure 1b). Since the composite hydrogel **NaPPDT**/**PAPA** with the composition 1/2 (*w*/*w*) did not show a gel state, the following measurements were performed to analyze only the composite hydrogel **NaPPDT**/**PAPA** 1/1 (*w*/*w*). The composite hydrogels maintained their homogeneous gel state for at least 1 year.

**Figure 2:**
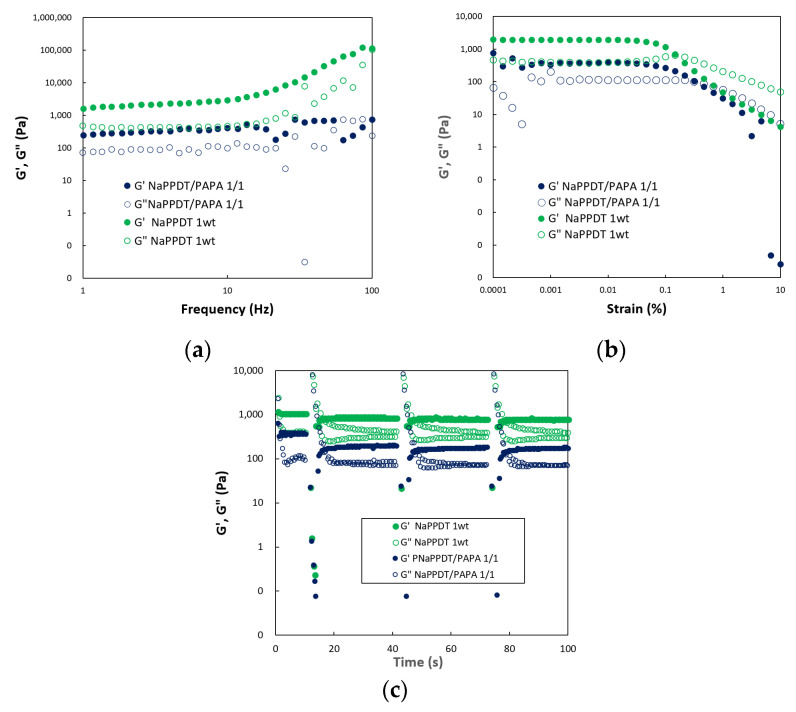
Rheometric results of **NaPPDT**/**PAPA** hydrogel systems: (**a**) frequency sweep, (**b**) strain sweep, and (**c**) periodic step-shear test (a vibration of G” was observed in both samples, probably due to the vibration of gels while the measurements were taken).

To investigate the **NaPPDT**/**PAPA** composite’s hydrogel state, a qualitative evaluation of the mechanical properties of the gels was performed with rheometric measurements (Figure 2a,b). By applying the frequency sweep to the gels (Figure 2a), the existence of the pseudo plateau, where the magnitude of the modulus showed that the storage modulus (G′) > the loss modulus (G″), proved that gelation had occurred, as has been shown in other organic polymer gels [57]. By increasing the stress on the gels (Figure 2b), G′ and G″ demonstrated the transition from the gel state (G′ > G″) to the sol state (G′ < G″) over the intersection of G′ and G″. The results of the composite hydrogel also confirmed the gel state and the formation of a softer and more tenacious hydrogel than the corresponding **NaPPDT** hydrogel, evidenced by the shift in the value and cross point of G′ and G″, as also indicated for the other mixed composite hydrogels composed of LAPONITE^®^ [53] or polyaniline/water-soluble polymer complex with **NaPPDT** [54].

**Figure 3:**
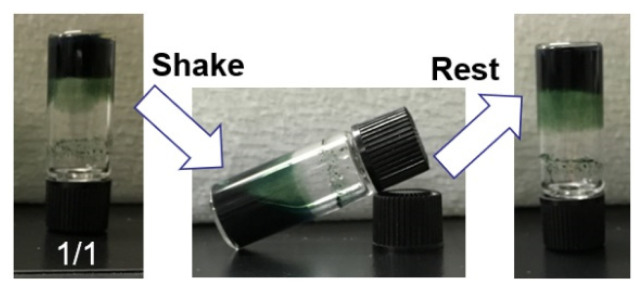
Photos of thixotropic behavior of 1.0 wt% **NaPPDT**/1.0 wt% **PAPA** aq. 1/1 (*w*/*w*) in a vial: (**left**) before shaking (gel state), (**center**) immediately after shaking (sol state), and (**right**) after resting for 1 min.

Additionally, as shown in Figure 2c and Figure 3, the composite hydrogel **NaPPDT**/**PAPA** 1/1 (*w*/*w*) showed thixotropic behavior similar to the **NaPPDT** hydrogel in a single component. This may suggest that mixing **NaPPDT** with **PAPA** improves the gel-forming abilities of the polymer hydrogelator **NaPPDT**, as previously shown in the other composite hydrogels [45,46]. The quantitative data on the composite hydrogel’s thixotropic behavior were also measured by step-shear measurements after repeatedly applying a large deformation force and resting (Figure 2c). It was demonstrated that after applying the deformation force, there was a repeated recovery from the sol state (G’ < G”) to the gel state (G’> G”) at the same rate each time in this system [58]. Recovery from gel to sol took 1 min using the inversion method but only a few tens of seconds using rheometry, since we were observing the behavior in a tiny space with a gap of 0.5 mm. The obtained results for the **NaPPDT** hydrogel and the composite hydrogel suggested that the thixotropic behavior of the composite hydrogel was the same as that of the **NaPPDT** hydrogel, whereas adding **PAPA** enhanced the gel properties of the composite hydrogel and softened the obtained composite.

**Figure 4:**
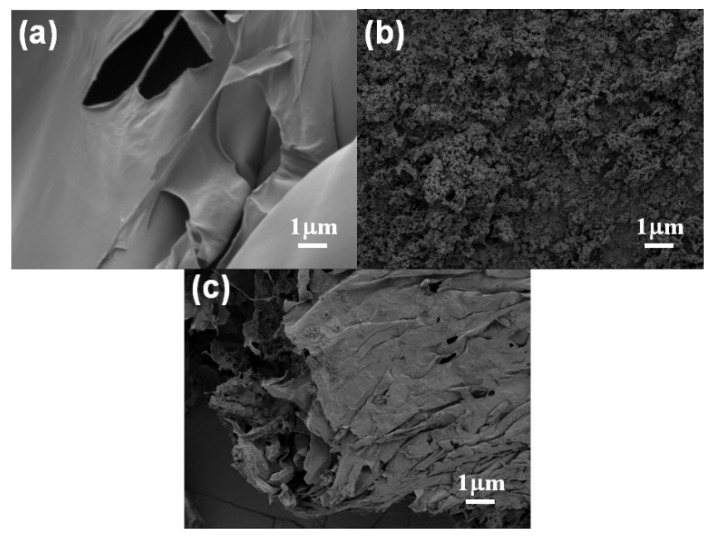
Scanning electron microscopy (SEM) images of xerogels and the dried sample obtained from freeze-drying method: (**a**) **NaPPDT** xerogel prepared from the corresponding **NaPPDT** 1.0 wt% hydrogel, (**b**) dried sample of 1.0 wt% **PAPA** aq., (**c**) **NaPPDT**/**PAPA** composite xerogel prepared from the corresponding **NaPPDT**/**PAPA** 1/1 (*w*/*w*) composite hydrogel.

Scanning electron microscopy (SEM) was performed for freeze-dried samples of **PAPA** aq. and hydrogels (xerogels) to closely observe the composite hydrogel microstructure. As shown in Figure 4, while the composite’s components were in the order of dozens of nanometers of fiber, the components of the **PAPA** particles were aggregated in the order of micrometers, and the components of the **NaPPDT** displayed porous wrinkled networks also in the order of micrometers [52]. The structure of the composite xerogel seemed to comprise a network of components (fibers) that were finer than those of the freeze-dried **PAPA** and **NaPPDT** xerogel. This may suggest that the presence of **PAPA** prevents the aggregation of the **NaPPDT** polymer chains from creating thicker network components. This suppression of **NaPPDT** aggregation may be related to the increased strain value of the sol–gel transition, as the mesh density is enhanced while the overall polymer concentration is diluted and the gel becomes soft. Although the freeze-drying process may influence the maintenance of the gel state network structure by drying-induced aggregation and ice growth [59], these SEM images nevertheless reflect the difference in the network structure of a native network of gels.

**Figure 5:**
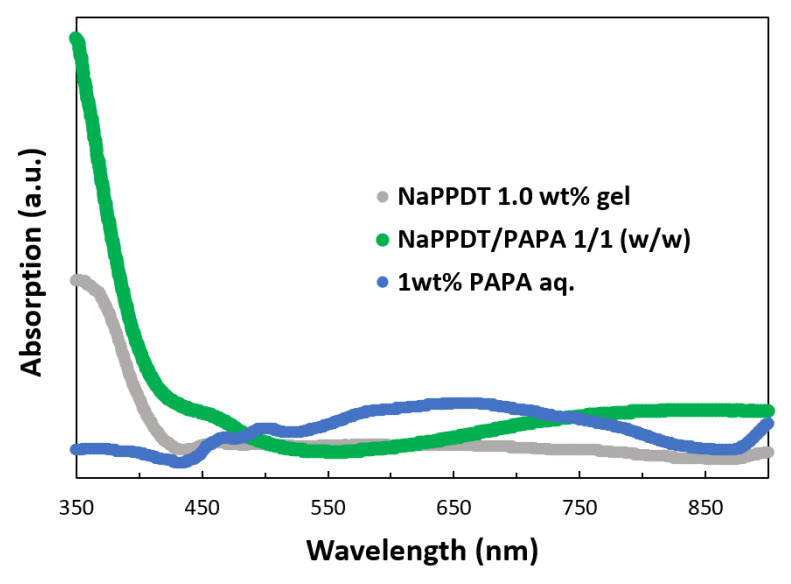
The UV-vis absorption bands of 1.0 wt% **NaPPDT**, **NaPPDT**/**PAPA** 1/1 (*w*/*w*) composite hydrogel, and 1.0 wt% **PAPA** aq.

The electrical conductivity of the composite hydrogel was determined to explore its potential application as an electroactive material in electrochemical devices. As polyanilines show their electric conductivity in the doping state, the doping state of **PAPA** was checked by investigating the existence of doping (the existence of radical cation species) with the UV-vis spectrum of the **NaPPDT**/**PAPA** composite hydrogel (Figure 5). The absorption band of the composite, ranging from 650 to 900 nm, was a typical band of doped polyaniline derivatives and was attributed to the emeraldine-oxidized state of polyanilines [60,61]. It is suggested that the electronic environment of **PAPA** was changed by the existence of **NaPPDT**, and this result showed some interaction between **PAPA** and **NaPPDT** (unfortunately, no specific peaks or shifts were observed in the FT-IR measurements).

By applying the four-probe method of electronic conductivity measurement to the **NaPPDT**/**PAPA** 1/1 (*w*/*w*) composite hydrogels, we found that the electronic conductivity was 2.5 × 10^−5^ S cm^−1^, which is within the range of semiconducting polymers and superior to our previous gelator/polyaniline composite hydrogel [54]. Although this value is relatively low for conducting polymers due to the existence of pure water, and it is in the same range of conductivity as the composite hydrogels consisting of **NaPPDT** and polyaniline ion complexes [54], it maintains a sufficient current for the electrochemical process, as was shown in poly(*N*-vinylcarbazole) with a conductivity of 10^−6^ S cm^−1^, which functioned as an electroactive and electrode-active material [62,63].

**Figure 6:**
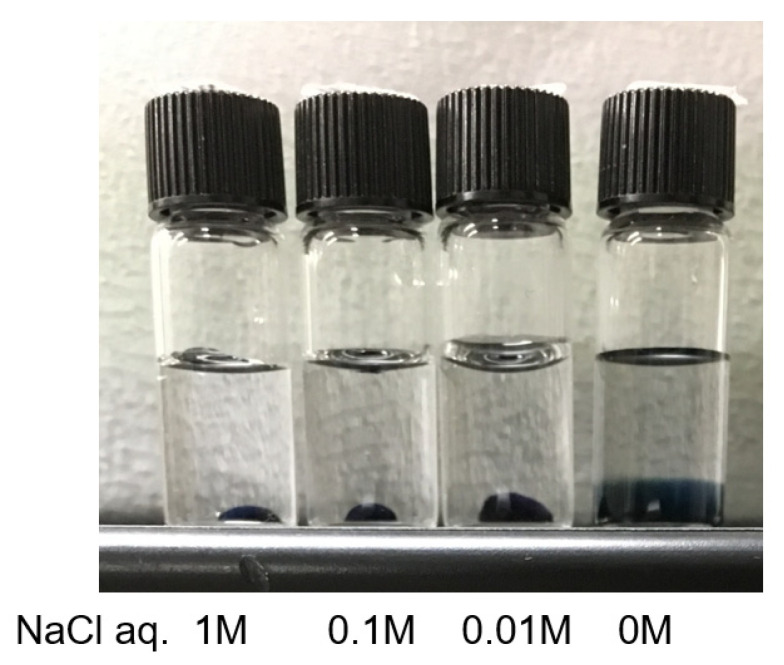
Solubility test of the hydrogels using a lump of the **NaPPDT**/**PAPA** 1/1 (*w*/*w*) composite hydrogel in NaCl aq. after 1 week.

**Figure 7:**
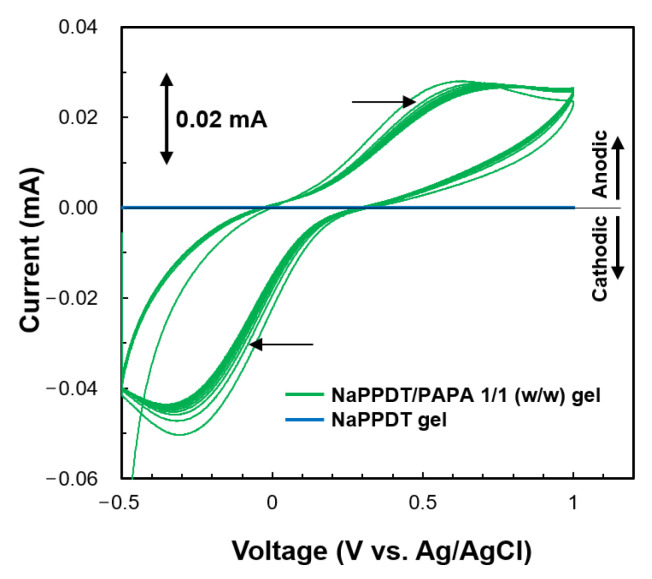
Cyclic voltammograms of hydrogels in 0.1 NaCl aq.: **NaPPDT**/**PAPA** 1/1 (*w*/*w*) composite gel and 1.0 wt% **NaPPDT** gel (10 oxidation/reduction cycles, scan rate: 0.05 V s^−1^). The horizontal arrows indicate the direction of movement of the curve as the number of scans increases.

Finally, the electrochemical response of the composite gels was examined to verify the potential electrochemical properties of the **NaPPDT**/**PAPA** composite hydrogels in a thin-layer cell. Before the electrochemical measurements, it was observed that the gel state was stable, as shown in Figure 6, and the **NaPPDT**/**PAPA** hydrogel solubility in water was suppressed in NaCl electrolyte aqueous solution, indicating a pseudo-chemically reversible electrochemical process of recycling potential sweeps (Figure 7). As shown in Figure 7, whereas the **NaPPDT** gel showed smaller and inert electrochemical responses, the **NaPPDT**/**PAPA** 1/1 (*w*/*w*) composite hydrogel in a thin-layer cell as a working electrode showed an oxidation peak at 0.60 V in the anodic oxidation process and a corresponding smaller reduction peak at −0.30 V in the cathodic oxidation process, attributed to the electrochemical response of the polyaniline complex in the composite hydrogel. These electrochemical processes were similar to those of a typical polyaniline film, as seen in the literature [61,64]. These results demonstrate that the composite gels may become potentially paintable and thixotropic electrode-active materials for electrochemical devices. To utilize the paintable and thixotropic properties of the composite gels, further electrochemical evaluation of the composite gel electrode is required.

## 3. Conclusions

In conclusion, the simple mixing of a water-soluble electronically conducting polyaniline complex **PAPA** and the polymeric hydrogelator **NaPPDT** produced a new composite molecular hydrogel with improved gelation ability and thixotropic behavior, although no individual component exhibited these gel properties in the same concentration regions. The new composite hydrogel showed the retention of the gel state in an electrolyte solution and electronic conductivity in a semiconducting region, which proves the applicability of electroactive materials in electrochemical devices. The **NaPPDT**/**PAPA** 1/1 (*w*/*w*) composite hydrogels demonstrated electrochemical responses owing to the polyaniline complex in an aqueous electrolyte. These results showed that composite hydrogels might be suitable for new potential paintable electrode materials. A detailed examination of the electrochemical properties of the composite hydrogels for glucose sensors is ongoing.

## 4. Materials and Methods

**NaPPDT** (M_n_ = 10,000) was synthesized according to the literature procedures [52,65,66]. Aniline (99%, Wako Pure Chemical Industries, Ltd., Tokyo, Japan) was used without further purification. All other chemicals were purchased from Wako Pure Chemical Industries, Ltd. and used without further purification. Water was deionized with an Elix UV 3 Milli-Q integral water purification system (Nihon Millipore K.K., Tokyo, Japan). Glucose oxidase from *Aspergillus niger* (Type VII, lyophilized powder, ≥100,000 units/g solid (without added oxygen)) was purchased from Sigma-Aldrich and used as received.

The water-soluble polyaniline complex (**PAPA**) was prepared via the oxidative polymerization of aniline with potassium persulfate (APS, electrophoresis grade) in the presence of phosphoric acid (PA). Firstly, in a sample tube, 470 mg aniline (5.00 mmol) and 1.65 g PA (15.00 mmol) were dissolved in 2 mL of Triton-X-100 1 wt% aqueous solution (solution A). In another sample tube, 582 mg APS (2.50 mmol) was dissolved in 2 mL of Triton-X-100 1 wt% aqueous solution (solution B). Solutions B and A were placed in a refrigerator (5 °C) for 5 min, and then solution B was added to solution A in an ice bath (10 °C) and allowed to react for 10 min without stirring. The mixed solution immediately turned black/green and solidified. The mixed solution was left to stand at room temperature for 24 h and then placed in a Fisherbrandt regenerated cellulose dialysis tube (molecular weight cut off: 3500 Da, Fisher Scientific and wexer lifescience Co.,Ltd., Tokyo, Japan) with pure water. The concentrated **PAPA** aqueous solution after dialysis was prepared (based on the weight of the solution after it was dried) so that the dry matter weight was 1.0 wt% in the aqueous solution. Unfortunately, the dried samples from the **PAPA** dispersion were insoluble in D_2_O and DMSO-*d_6_*, so the chemical structure could not be measured by NMR measurements.

Elemental analysis result for **PAPA** was performed with YANACO CHN CORDER MT-5 (organic trace element analyzer, YANACO technicalscience, Co., Ltd., Tokyo, Japan). Elemental analysis result for **PAPA**: Found: C, 62.38; H, 4.37; N, 11.95 (1st measurement) and Found: C, 62.61; H, 4.40; N, 11.95 (2nd measurement). From the elemental analysis of **PAPA**, the molar mass ratio of the aniline moiety (An: -NHC_6_H_4_-) to the phosphate moiety (PA: P=O(OH)_3_), taking into account the presence of bound water, yields the following ratio: An/PA/H_2_O = 100/25/5 = 20/5/1 (C, 61.85; H, 5.09; N, 12.02).

The preparation of **NaPPDT**/**PAPA** composite hydrogels was done as follows: at first, 1.0 wt% **NaPPDT** hydrogel was made by mixing **NaPPDT** solid and pure water and rest for one day at room temperature. Then 1.0wt% **PAPA** aq. was added to the 1.0 wt% **NaPPDT** gel at room temperature and mixed by use of a vortex genie (Scientific Industries, Inc., Bohemia, New York, NY, USA). Before measurements, the mixed composite hydrogels were rested for 30 min.

Gelation ability and thixotropic behavior were evaluated by the vial inversion method (a method in which a vial containing gel is inverted and judged to be a gel state if it does not drip). In evaluating thixotropic behavior, if it recovered from a sol to a gel when it was broken with a vortex gene to form a sol and inverted after standing for a certain time (If it is not recovered and sol, it will drip).

SEM image measurements were carried out using a SU-8000 scanning electron microscope (Hitachi High-Technologies Corporation, Tokyo, Japan) at 1.0 kV. The freeze-dried sample or freeze-dried hydrogels (xerogels) were measured on a conductive tape on the brass SEM stage. The samples were coated with a spattering of Pt (10 nm-thick) to add electrical conductivity.

Rheological measurements were performed using an MCR-301 rheometer (Anton Paar Japan K.K., Tokyo, Japan) with a parallel plate (8 mm diameter) at a gap of 0.50 mm at 25 °C. The frequency sweeps were carried out with γ of 0.01 %. The strain sweeps were carried out with a constant angular frequency of 1 rad s^−1^. The repeated step-shear measurements were carried out with a normal strain with an amplitude of 0.01% and frequency of 1 Hz and a large strain with a shear rate of 3000 s^−1^ for 0.1 s.

Measurements of the absorbance of a 0.4 mm thick polymer or hydrogel sample between slide glasses with overlapped Kapton films (0.4 mm thickness) as a spacer and **PAPA** aq. in a quartz cuvette with a light path length of 1.0 mm were performed using a measurement system consisting of a spectrometer HR4000 (Ocean Optics, Inc., Tokyo, Japan), UV-VIS-NIR light source DH-200-BAL (Mikropack GmbH), and variable attenuator FVA-UV (Ocean Optics, Inc., Tokyo, Japan) controlled by PC software OPwave (Ocean Photonics, Tokyo, Japan). The measurement system was constructed by Ocean Photonics.

The electronic conductivity of the composite hydrogel was evaluated with a four-probe method using an interdigitated array electrode (BAS Inc., Tokyo, Japan), a digital multimeter 34401a (KEYSIGHT TECHNOLOGIES, Santa Rosa, CA, USA) and (ADC CORPORATION, Saitama, Japan), a digital multimeter GDM 8255 (Good Will Instrument Co., Ltd., New Taipei City, Taiwan), and a DC power supply U8001 A (30 V/3 A, KEYSIGHT TECHNOLOGIES, Santa Rosa, CA, USA). An interdigitated array electrode for the four-probe method was placed on a cool plate CP-085 (SANSYO, Co., Ltd., Tokyo, Japan) at 25 °C. The current value between the inner electrodes was measured when a DC voltage was applied between the outer electrodes of the four terminals, and the resistance of the gel sample was calculated from the distance between the electrodes and the electrode area. We placed the gel on the electrode, and after an ionic current of the composite hydrogel was settled, the electronic conductivity of the hydrogel was measured.

For electrochemical measurement using an electrochemical analyzer ALS model 6271E (BAS Inc., Tokyo, Japan), the composite gel or **NaPPDT** gel was sandwiched between an indium–tin–oxide electrode (10 ohm/square) and a glass slide via spacers (overlapped Kapton films: 0.4 mm thickness) to form a thin-layer cell (active area: 9 mm × 10 mm). The protruded gel that formed during the fabrication of the cell was wiped off. Cyclic voltammetry for the thin-layer cell made with the gel immersed in NaCl aq. (0.1 mol dm^−3^) was conducted with platinum wire and Ag/AgCl (3 mol dm^−^^3^) as the counter and reference electrodes, respectively. To prepare for future electrochemical measurements, measurements were made on a composite gel containing 1 wt% glucose oxidase.
